# EARLY MULTIDIMENSIONAL MOBILITY ASSESSMENTS FOR DISCRIMINATING DISCHARGE-LEVEL AMBULATION AND FUNCTIONAL INDEPENDENCE IN SUBACUTE STROKE INPATIENTS: A RETROSPECTIVE COHORT STUDY

**DOI:** 10.2340/jrm.v58.45503

**Published:** 2026-04-16

**Authors:** Seung Heun AN, Eun Joo KIM, Jun Min LEE

**Affiliations:** 1 Department of Gait Lab, National Rehabilitation Center, Seoul, South Korea; 2 Department of Rehabilitation Medicine, Rehabilitation Hospital, National Rehabilitation Center, Seoul, South Korea

**Keywords:** postural balance, gait, rehabilitation, stroke, mobility limitation, outcome assessment, healthcare

## Abstract

**Objective:**

To examine whether early multidimensional mobility assessments discriminate independent ambulation and functional independence on discharge in subacute stroke inpatients with supervised walking ability.

**Design:**

Retrospective cohort study.

**Subjects/Patients:**

Fifty subacute stroke inpatients (≤ 5 months post-stroke).

**Methods:**

Independent ambulation and functional independence were defined as Functional Ambulation Category ≥ 4 and Modified Barthel Index 75 on discharge, respectively. Discriminative ability of admission assessments was evaluated using univariate binary logistic regression and receiver operating characteristic curve analysis.

**Results:**

The Berg Balance Scale and the modified Four Square Step Test demonstrated the highest discriminative performance. For independent ambulation, the Berg Balance Scale (≥ 40.5) yielded an area under the curve of 0.74 (95% confidence interval: 0.60–0.88) with 82% accuracy, and the modified Four Square Step Test (≤ 31.52 s) yielded an area under the curve of 0.78 (0.64–0.91) with 80% accuracy. For functional independence, the Berg Balance Scale (≥ 42.5) yielded an area under the curve of 0.74 (0.60–0.88) with 74% accuracy, and the modified Four Square Step Test (≤ 32.88 s) yielded an area under the curve of 0.71 (0.57–0.86) with 70% accuracy.

**Conclusion:**

Early balance and multidirectional stepping performance may be useful for screening to support goal-setting and discharge planning.

## LAY ABSTRACT

Regaining independent walking and functional independence is a key goal of stroke rehabilitation. Early identification of patients who are likely to achieve these outcomes can support goal setting and discharge planning. This study examined whether mobility and balance assessments performed shortly after admission could distinguish patients who achieved independent walking and functional independence by discharge. In 50 subacute stroke inpatients who required supervised walking on admission, the Berg Balance Scale and the modified Four Square Step Test showed the strongest ability to distinguish discharge-level outcomes among commonly used assessments. These balance-based, multidirectional mobility tests may serve as practical screening tools to support rehabilitation planning and discharge decision-making in subacute stroke inpatient rehabilitation.

Stroke is a leading neurological condition associated with long-term disability, and loss of walking ability is a major determinant of reduced autonomy and quality of life (1, 2). Diminished ambulatory independence limits activities of daily living and increases reliance on others (3), with substantial implications for independent living and community reintegration after discharge. Accordingly, the goal of stroke rehabilitation extends beyond recovery of isolated physical functions to restoration of independent ambulation and functional independence (4). The subacute phase, within 1–6 months post-stroke in inpatient rehabilitation, represents a key recovery period during which functional gains are often most pronounced, and early functional status has been regarded as an important clinical indicator related to subsequent outcomes (5, 6).

Multidimensional mobility assessments capturing balance, stepping ability, and functional movement performance may be especially relevant in subacute stroke inpatients with supervised walking ability, particularly those classified as Functional Ambulation Category (FAC) level 3 on admission, as these measures reflect task demands required to transition toward independence on discharge (7–9). In clinical practice, standardized tools are commonly used to evaluate walking speed and endurance, functional mobility, balance, and multidirectional stepping, including the 10-m walk test (10mWT), 6-min walk test (6MWT), Timed Up and Go test (TUG), Berg Balance Scale (BBS), and modified Four Square Step Test (mFSST) (10–16). However, despite their widespread use, evidence remains limited regarding the extent to which admission assessment results can discriminate clinically meaningful discharge outcomes, such as independent ambulation (FAC ≥ 4) and functional independence (Modified Barthel Index [MBI] ≥ 75). In addition, clinically applicable cut-off values to support routine decision-making have not been clearly established in this patient group (17).

Previous work has suggested that balance-related measures may be informative for identifying ambulation outcomes in subacute stroke, but studies have often examined a limited set of indicators (17). Few studies have integrated multiple admission assessments to simultaneously discriminate both independent ambulation and functional independence on discharge. Therefore, this retrospective cohort study analysed a set of standardized admission mobility assessments in subacute stroke inpatients and examined their ability to discriminate independent ambulation (FAC ≥ 4) and functional independence (MBI ≥ 75) on discharge (18). The objectives were to: (*i*) examine associations between admission assessments and discharge outcomes; (*ii*) evaluate discriminative validity and identify clinically applicable cut-off values; and (*iii*) examine the clinical usefulness of measures demonstrating superior discriminative performance.

## METHODS

### Study design and participants

This retrospective cohort study was conducted at the Gait Laboratory of the National Rehabilitation Center, Seoul, South Korea, using routinely collected Electronic Medical Records (EMR) and Gait Laboratory databases. Reporting followed the STROBE (Strengthening the Reporting of Observational Studies in Epidemiology) guidelines.

Participants were identified from inpatient records of individuals admitted for stroke rehabilitation within 5 months post-stroke who were classified as FAC level 3 on admission. FAC level 3 indicates the ability to walk indoors under supervision. This subgroup represents a transitional stage in which patients commonly progress toward independent ambulation during inpatient rehabilitation.

Inclusion criteria were: (*i*) sufficient cognitive function to understand and follow test instructions, defined as a Mini-Mental State Examination–Korean version (MMSE-K) score ≥ 24; (*ii*) medical stability after acute stroke management; and (*iii*) the ability to walk at least 10 m with or without a walking aid. Exclusion criteria were: (*i*) lower-limb orthopaedic conditions interfering with functional assessment; (*ii*) concomitant neurological disorders other than stroke; and (*iii*) recurrent stroke or bilateral hemiparesis.

Between July 2023 and May 2024, 63 patients were initially screened. Thirteen patients were excluded due to missing baseline assessment data or not meeting the admission FAC level 3 criterion (*n* = 7), having severe cognitive impairment (*n* = 3), or recurrent stroke (*n* = 3). Consequently, 50 participants were included in the final analysis. Missing data were handled using listwise deletion. To minimize selection bias, all eligible patients during the study period were consecutively included.

The mean time since stroke onset was 3.64 (SD 0.59) months (range 2–5 months). Baseline assessments were performed within 3 days of admission (index date), and discharge assessments were conducted approximately 4 weeks after admission. Independent ambulation and functional independence on discharge were defined as an FAC score ≥ 4 and an MBI score ≥ 75, respectively.

All mobility and gait assessments were performed using standardized protocols in a controlled environment. Demographic and clinical characteristics (age, sex, time since stroke onset, stroke type, affected side, and MMSE-K score) were extracted from EMR. Functional assessments collected on admission and discharge included FAC, MBI, 10mWT, 6MWT, 5-Times STST, TUG, F8WT, BBS, and mFSST.

Ethical approval for the study protocol was obtained from the Institutional Review Board of the National Rehabilitation Center (IRB No. NRC-2024-04-027). Written informed consent was waived owing to the retrospective study design. All procedures were conducted in accordance with the Declaration of Helsinki.

### Outcome measures

The primary outcomes of this study were independent ambulation and functional independence on discharge. FAC and MBI were selected as clinically interpretable indicators reflecting discharge-level mobility and activities of daily living independence.

FAC is a 6-point ordinal scale (0–5) that evaluates walking ability based on the level of assistance required during ambulation, regardless of the use of walking aids. An FAC score of 4 indicates independent walking on level surfaces without physical contact or supervision. The FAC has demonstrated excellent inter-rater reliability in individuals with stroke, with a reported kappa coefficient of 0.95 (19).

MBI is a standardized measure used to quantify performance in activities of daily living, comprising 10 items related to personal hygiene, bathing, feeding, bowel and bladder control, transfers, and mobility. Each item is scored on a weighted scale ranging from 0–5 or 0–15, yielding a total score from 0 to 100, with higher scores indicating greater functional independence. Functional levels are commonly classified as follows: total dependence (0–24), severe dependence (25–49), moderate dependence (50–74), mild dependence (75–90), minimal dependence (91–99), and complete independence (100) (20). The MBI has demonstrated excellent inter-rater reliability in neurological rehabilitation populations, including stroke, with a reported ICC of 0.98 (21).

### Assessment variables

The main assessment variables included the 10mWT and 6MWT to evaluate walking speed and endurance; the 5-Times STST to assess lower-extremity strength and functional performance; the TUG and F8WT to evaluate functional mobility; and the BBS and mFSST to assess balance performance and fall risk. All assessments were administered using identical procedures on admission (baseline) and on discharge.

The 10mWT was performed over a 14-m walkway, with walking speed (m/s) calculated from the time required to traverse the central 10 m, excluding acceleration and deceleration phases. Participants walked at a comfortable pace, and the mean of 3 trials was used. The 10mWT has demonstrated excellent test–retest reliability in individuals with stroke (ICC = 0.93) (22).

The 6MWT was conducted on a 30-m level corridor. Participants walked back and forth for 6 min and were instructed to cover as much distance as possible. Encouragement was provided, and the total distance walked was recorded. The 6MWT has demonstrated excellent inter-rater reliability in stroke populations (ICC = 0.95) (23).

The 5-Times STST was performed on a chair with a backrest and no armrests. Participants stood up and sat down 5 times as quickly as possible without using their upper limbs, and the total time was recorded. Inter-rater reliability has been reported as excellent (ICC = 0.999) (24).

The TUG was used to evaluate functional mobility and balance. Participants rose from a chair, walked 3 m, turned around, returned, and sat down. The mean of 3 trials was used. The TUG has demonstrated excellent test–retest reliability (ICC = 0.96) (25).

The F8WT evaluates turning ability and gait adaptability by measuring the time required to walk along a figure-of-eight pathway around 2 obstacles. In individuals with stroke, inter-rater and test–retest reliability have been reported as excellent (ICC = 0.94–0.99) (26).

The BBS consists of 14 items involving sitting, standing, and postural transitions. Each item is scored from 0 to 4, yielding a maximum total score of 56, with higher scores indicating better balance performance. Inter-rater reliability has been reported as excellent (ICC = 0.95) (27).

The mFSST evaluates multidirectional mobility, balance control, and fall risk by stepping across 4 quadrants arranged in a cross-configuration in clockwise and counter-clockwise directions. After 1 practice trial, 2 recorded trials were performed, and the faster time was used. Inter-rater reliability has been reported as excellent (ICC = 0.94) (16). All assessments were performed while participants wore their usual footwear.

These 7 measures were selected to capture complementary mobility domains in subacute stroke inpatients and to ensure safety and feasibility for patients with supervised walking ability on admission (FAC level 3). The battery was intended to support discrimination of functional transitions at discharge (FAC ≥ 4; MBI ≥ 75) and to compare discriminative performance across domains.

### Statistical analysis

All statistical analyses were performed using SPSS Statistics version 21.0 (IBM Corp, Armonk, NY, USA). Normality of continuous variables was assessed using the Shapiro–Wilk test. Participant characteristics were summarized as frequencies and percentages, and functional performance variables were presented as means (SD).

Associations between admission assessment variables (10mWT, 6MWT, 5-Times STST, TUG, F8WT, BBS, and mFSST) and discharge outcomes (FAC ≥ 4; MBI ≥ 75) were explored using Spearman’s rank correlation analysis as an exploratory approach to examine overall monotonic relationships between variables.

To exploratorily evaluate the discriminative ability of admission assessments for discharge outcomes, univariable binary logistic regression analyses were performed. Admission scores were entered as independent variables, and discharge independent ambulation (FAC ≥ 4) and functional independence (MBI ≥ 75) were analysed in separate models as dependent variables. For each model, B, OR with 95% CI, and Nagelkerke R² were reported. Because high intercorrelations among assessment variables indicated multicollinearity, multivariable regression analyses were not conducted.

To control for type I error inflation due to multiple comparisons, FDR adjustment using the Benjamini–Hochberg procedure was applied, and adjusted *p*-values were reported (28). Statistical significance was determined at an FDR-adjusted α level of 0.05.

Discriminative validity for discharge outcomes was also evaluated using receiver operating characteristic (ROC) analyses. Sensitivity, specificity, PPV, NPV, and accuracy were calculated for each measure. The AUC was used as an index of overall discriminative performance, with AUC ≥ 0.70 considered indicative of clinically acceptable discrimination (29).

Optimal cut-off values were determined using Youden’s index to balance sensitivity and specificity. Identified cut-offs were interpreted as clinically informative screening thresholds for classifying patients with discharge independent ambulation (FAC ≥ 4) or functional independence (MBI ≥ 75), rather than as definitive diagnostic criteria. Because PPV and NPV are influenced by outcome prevalence, these indices were interpreted within clinical contexts similar to the present subacute inpatient stroke cohort.

## RESULTS

### General characteristics and functional assessments of participants

A total of 50 participants with subacute stroke were included. Of these, 26 (52%) were men and 24 (48%) were women. The mean age was 60.88 (SD 12.47) years, and the mean time since stroke onset was 3.64 (SD 0.59) months. The stroke subtype was cerebral infarction in 31 participants (62%) and intracerebral haemorrhage in 19 (38%). Left-sided hemiparesis was present in 22 participants (44%) and right-sided hemiparesis in 28 (56%). The mean MMSE-K score was 29.26 (SD 1.67).

On admission, all participants were classified as FAC level 3 (100%). On discharge, 24 participants (48%) remained at FAC level 3, 25 (50%) progressed to FAC level 4, and 1 (2%) reached FAC level 5; 26 participants (52%) achieved independent ambulation (FAC ≥ 4). Admission and discharge functional assessment results are summarized in Table I.

**Table I:** General characteristics and functional assessments of participants (*n* = 50)

Variables	N (%) or Mean ± SD (Min–Max)	
Gender, male/female, *n* (%)	26 (52)/24 (48)	
Age, year, mean~SD (range)	60.88±12.47 (35–82)	
Onset, months, mean~SD (range)	3.64±0.59 (2–5)	
Diagnosis, infarction/haemorrhage, *n* (%)	31 (62)/19 (38)	
Paretic side, left/right, *n* (%)	22 (44)/28 (56)	
MMSE-K, score, mean~SD (range)	29.26±1.67 (24–30)	
Functional assessments	Initial assessments	Discharge assessments
Variables, mean±SD/median (IQR)		
FAC (score)	FAC 3 = 50 (100%)	FAC 3 = 24 (48%) FAC 4 = 25 (50%) FAC 5 = 1 (2%)
MBI (score)	65.16±12.70/68 (58.50–72)	77.68±11.97/77.5 (71.75–86.25)
10mWT (m/s)	0.41±0.15/0.40 (0.27–0.54)	0.55±0.21/0.54 (0.37–0.69)
6MWT (m)	131.20±43.36/129.25 (94.75–158)	163.96±65.36/146.50 (110–219.15)
5-Times STST (sec)	23.27±8.88/21.21 (16.15–27.92)	16.76±6.75/15.79 (11.92–19.39)
TUG (sec)	30.81±12.17/27.56(22.10–36.39)	24.11±10.85/20.70 (16.30–29.80)
F8WT (sec)	27.39±9.90/24.99 (20.05–32.46)	21.49±9.27/18.56 (14.23–26.97)
BBS (score)	38.34±3.80/39 (36–41)	46.88±3.99/47.50 (44–49.25)
mFSST (sec)	41.77±11.42/40.44 (33.49–50.18)	31.59±10.76/28.81 (22.50–40.24)

SD: standard deviation; MMSE-K: Mini Mental State Examination-Korean version; FAC: Functional Ambulation Category; MBI: Modified Barthel Index; 10mWT: 10-meter Walk Test; 6MWT: 6-minute Walk Test; 5-Times STST: Five-Times Sit-to-Stand Test; TUG: Timed Up and Go test; F8WT: Figure-of-8 Walk Test; BBS: Berg Balance Scale; mFSST: Modified Four Square Step Test.

### Correlations between admission functional assessment scores and discharge FAC and MBI

Discharge FAC demonstrated positive correlations with 10mWT (*r* = 0.44, *p* < 0.05) and 6MWT (*r* = 0.40, *p* < 0.05), and negative correlations with TUG (*r* = −0.41, *p* < 0.05) and F8WT (*r* = −0.42, *p* < 0.05). The strongest correlations with FAC were observed for BBS (*r* = 0.70, *p* < 0.01) and mFSST (*r* = −0.63, *p* < 0.01). Similarly, Discharge MBI was positively correlated with 10mWT (*r* = 0.42, *p* < 0.05) and 6MWT (*r* = 0.47, *p* < 0.05), and negatively correlated with TUG (*r* = −0.39, *p* < 0.05) and F8WT (*r* = −0.40, *p* < 0.05). The strongest correlations with MBI were also observed for BBS (*r* = 0.63, *p* < 0.01) and mFSST (*r* = −0.61, *p* < 0.01). In contrast, 5-Times STST was not significantly correlated with either FAC or MBI (Table II).

**Table II:** Correlations (Spearman’s rho) between admission functional assessment scores and discharge outcomes: Independent ambulation (FAC ≥ 4) and functional independence (MBI ≥ 75) on discharge (*n* = 50)

Variables	10mWT	6MWT	5-Times STST	TUG	F8WT	BBS	mFSST
FAC	ρ = 0.44^*^	ρ = 0.40^*^	ρ = –0.13	ρ = –0.41^*^	ρ = –0.42^*^	ρ = 0.70^**^	ρ = –0.63^**^
MBI	ρ = 0.42^*^	ρ = 0.47^*^	ρ = –0.15	ρ = –0.39^*^	ρ = –0.40^*^	ρ = 0.63^**^	ρ = –0.61^**^

FAC: Functional Ambulation Category; MBI: Modified Barthel Index; 10mWT: 10-meter Walk Test; 6MWT: 6-minute Walk Test; 5-Times STST: Five-Times Sit- to-Stand Test; TUG: Timed Up and Go test; F8WT: Figure-of-8 Walk Test; BBS: Berg Balance Scale; mFSST: Modified Four Square Step Test.

* *p* < 0.05,

** *p* < 0.01.

### Univariable logistic regression analyses for discharge outcomes

Univariable binary logistic regression analyses were performed to examine associations between admission assessment scores and discharge outcomes, independent ambulation (FAC ≥ 4) and functional independence (MBI ≥ 75). All *p*-values were adjusted using the Benjamini–Hochberg FDR procedure, and adjusted *p*-values < 0.05 were considered statistically significant (Table III).

**Table III:** Univariable logistic regression analysis of admission functional assessment scores associated with independent ambulation (FAC ≥ 4) and functional independence (MBI ≥ 75) ont discharge (*n* = 50)

Independent variables	*B*	SE	Adjusted *p*-value	EXP (*B*)	95% CI	Nagelkerke R^2^
Associated with independent ambulation on discharge						
10mWT	0.01	0.01	0.025	1.01	1.00–1.02	0.23
6MWT	0.02	0.01	0.031	1.02	1.00–1.03	0.15
5-Times STST	–0.04	0.03	0.156	0.95	0.89–1.02	0.06
TUG	–0.06	0.03	0.032	0.94	0.89–0.99	0.15
F8WT	–0.08	0.04	0.028	0.92	0.85–0.98	0.18
BBS	0.27	0.10	0.019	1.31	1.07–1.61	0.23
mFSST	–0.10	0.03	0.021	0.91	0.85–0.97	0.27
Associated with functional independence on discharge						
10mWT	0.01	0.01	0.057	1.01	1.00–1.02	0.13
6MWT	0.02	0.01	0.049	1.01	1.00–1.03	0.14
5-Times STST	–0.03	0.03	0.405	0.97	0.91–1.04	0.02
TUG	–0.05	0.03	0.069	0.95	0.91–1.00	0.10
F8WT	–0.08	0.03	0.042	0.92	0.86–0.99	0.17
BBS	0.25	0.10	0.042	1.28	1.06–1.55	0.20
mFSST	–0.06	0.03	0.050	0.94	0.89–1.00	0.18

Adjusted *p*-values were calculated using the Benjamini–Hochberg false discovery rate method within each outcome model to control for multiple comparisons among admission assessment variables.

SE: standard error; EXP(B): odds ratio; CI: confidence interval; FAC: Functional Ambulation Category; MBI: Modified Barthel Index; 10mWT: 10-meter Walk Test; 6MWT: 6-minute Walk Test; 5-Times STST: Five-Times Sit-to-Stand Test; TUG: Timed Up and Go test; F8WT: Figure-of-8 Walk Test; BBS: Berg Balance Scale; mFSST: Modified Four Square Step Test. *p*-values < 0.05 were considered statistically significant.

For independent ambulation (FAC ≥ 4), admission 10mWT (OR = 1.01, 95% CI = 1.00–1.02), 6MWT (OR = 1.02, 95% CI = 1.00–1.03), and BBS (OR = 1.31, 95% CI = 1.07–1.61) were significantly and positively associated with the outcome (all adjusted p < 0.05). In contrast, admission TUG (OR = 0.94, 95% CI = 0.89–0.99), F8WT (OR = 0.92, 95% CI = 0.85–0.98), and mFSST (OR = 0.91, 95% CI = 0.85–0.97) were significantly inversely associated with independent ambulation (all adjusted *p* < 0.05). Admission 5-Times STST was not significantly associated with independent ambulation.

For functional independence (MBI ≥ 75), admission 6MWT (OR = 1.01, 95% CI = 1.00–1.03) and BBS (OR = 1.28, 95% CI = 1.06–1.55) were significantly and positively associated with the outcome (adjusted *p* < 0.05). In contrast, admission F8WT (OR = 0.92, 95% CI = 0.86–0.99) was significantly inversely associated with functional independence (adjusted *p* < 0.05). Admission 10mWT, TUG, and 5-Times STST were not significantly associated with functional independence, while mFSST showed a borderline non-significant association (adjusted *p* = 0.050) (see Table III).

### Receiver operating characteristic analyses

ROC analyses were conducted to evaluate the discriminative performance of admission mobility assessment scores for independent ambulation on discharge (FAC ≥ 4). Among the assessed measures, mFSST and BBS demonstrated superior discriminative performance. The mFSST yielded the highest AUC (0.78, 95% CI: 0.64–0.91), with an optimal cut-off value of ≤ 31.52 s (adjusted *p* < 0.007). The BBS also demonstrated acceptable discrimination (AUC = 0.74, 95% CI: 0.60–0.88), with an optimal cut-off value of ≥ 40.5 points (adjusted *p* < 0.014).

In addition, TUG (cut-off ≤ 22.5 s; AUC = 0.74, adjusted *p* < 0.014), F8WT (cut-off ≤ 24.03 s; AUC = 0.74, adjusted *p* < 0.014), 10mWT (cut-off ≥ 0.41 m/s; AUC = 0.72, adjusted *p* < 0.019), and 6MWT (cut-off ≥ 124.5 m; AUC = 0.70, adjusted *p* < 0.019) demonstrated statistically significant discriminative performance, with AUC values of 0.70 or higher. In contrast, 5-Times STST demonstrated limited discrimination (AUC = 0.57, 95% CI: 0.41–0.74) and was not statistically significant after FDR adjustment (adjusted *p* = 0.382).

ROC analyses for functional independence on discharge (MBI ≥ 75) indicated that BBS and mFSST provided stronger discriminative performance. The BBS yielded an AUC of 0.74 (95% CI: 0.60–0.88), with an optimal cut-off value of ≥ 42.5 points (adjusted *p* < 0.028). The mFSST demonstrated an AUC of 0.71 (95% CI: 0.57–0.86), with an optimal cut-off value of ≤ 32.88 s (adjusted *p* < 0.028).

Other measures, including 10mWT (cut-off ≥ 0.41 m/s; AUC = 0.70, adjusted *p* < 0.028), TUG (cut-off ≤ 23.11 s; AUC = 0.70, adjusted *p* < 0.028), F8WT (cut-off ≤ 23.48 s; AUC = 0.70, adjusted *p* < 0.028), and 6MWT (cut-off ≥ 126.5 m; AUC = 0.69, adjusted *p* < 0.032), also showed statistically significant discrimination. In contrast, 5-Times STST did not show significant discrimination for functional independence (AUC = 0.57, 95% CI: 0.40–0.73; adjusted *p* = 0.440) (Table IV).

**Table IV:** Receiver operating characteristic (ROC) analysis of admission mobility scores for discriminating discharge ambulation (FAC ≥ 4) and functional independence (MBI ≥ 75) (*n* = 50)

Variables	Cut-off value	AUC	SE	95% CI	Adjusted *p*-value
Discriminative performance for independent ambulation at discharge					
10mWT	≥ 0.41 m/s	0.72	0.07	0.57–0.86	0.019
6MWT	≥ 124.5 m	0.70	0.07	0.55–0.85	0.019
5-Times STST	–	0.57	0.08	0.41–0.74	0.382
TUG	≤ 22.5 s	0.74	0.07	0.59–0.88	0.014
F8WT	≤ 24.03 s	0.74	0.07	0.60–0.87	0.014
BBS	≥ 40.5 score	0.74	0.07	0.60–0.88	0.014
mFSST	≤ 31.52 s	0.78	0.07	0.64–0.91	0.007
Discriminative performance for functional independence on discharge					
10mWT	≥ 0.41m/s	0.70	0.08	0.56–0.85	0.028
6MWT	≥ 126.5m	0.69	0.08	0.54–0.84	0.032
5-Times STST	–	0.57	0.08	0.40–0.73	0.440
TUG	≤ 23.11 s	0.70	0.08	0.56–0.85	0.028
F8WT	≤ 23.48 s	0.70	0.08	0.55–0.85	0.028
BBS	≥ 42.5 score	0.74	0.07	0.60–0.88	0.028
mFSST	≤ 32.88 s	0.71	0.07	0.57–0.86	0.028

Cut-off values were determined using the Youden index. Measures with AUC < 0.70 were considered to have limited discriminative ability. Adjusted *p*-values were calculated using the Benjamini–Hochberg false discovery rate method.

AUC: area under the curve; SE: standard error; CI: confidence interval; FAC: Functional Ambulation Category; MBI: Modified Barthel Index; 10mWT: 10-meter Walk Test; 6MWT: 6-minute Walk Test; 5-Times STST: Five-Times Sit-to-Stand Test; TUG: Timed Up and Go test; F8WT: Figure-of-8 Walk Test; BBS: Berg Balance Scale; mFSST: Modified Four Square Step Test. *p*-values < 0.05 were considered statistically significant.

### Classification performance of admission mobility scores at ROC-derived cut-off values for discharge outcomes

Using the ROC-derived cut-off values, the classification performance of admission mobility assessment scores for discharge independent ambulation (FAC ≥ 4) and functional independence (MBI ≥ 75) was compared using sensitivity, specificity, PPV, NPV, and accuracy (Table V).

**Table V:** Classification performance of admission mobility scores at receiver operating characteristic (ROC)-derived cut-off values for discharge ambulation (FAC ≥ 4) and functional independence (MBI ≥ 75) (*n* = 50)

Variables	Cut-off value	FAC ≥ 4 (*n* = 26)	FAC < 4 (*n* = 24)	total	Se(%)	Sp(%)	PPV(%)	NPV(%)	Ac(%)
Classification performance for discharge ambulation									
10mWT	≥ 0.41 m/s	18	7	25	18/26 (69)	17/24 (71)	18/25 (72)	17/25 (68)	35/50 (70)
	< 0.41 m/s	8	17	25					
6MWT	≥ 124.5 m	18	9	27	18/26 (69)	15/24 (63)	18/27 (67)	15/23 (65)	33/50 (66)
	< 124.5 m	8	15	23					
TUG	≤ 22.5 s	15	8	23	15/26 (58)	16/24 (67)	15/23 (65)	16/27 (59)	31/50 (62)
	> 22.5 s	11	16	27					
F8WT	≤ 24.03 s	16	7	23	16/26 (62)	17/24 (71)	16/23 (70)	17/27 (63)	33/50 (66)
	> 24.03 s	10	17	27					
BBS	≥ 40.5 score	24	7	31	24/26 (92)	17/24 (71)	24/31 (77)	17/19 (89)	41/50 (82)
	< 40.5 score	2	17	19					
mFSST	≤ 31.52 s	18	2	20	18/26 (69)	22/24 (92)	18/20 (90)	22/30 (73)	40/50 (80)
	> 31.52 s	8	22	30					

Variables	Cut-off value	MBI ≥ 75 (*n* = 30)	MBI < 75 (*n* = 20)	total	Se(%)	Sp(%)	PPV(%)	NPV(%)	Ac(%)

Classification performance for functional independence									
10mWT	≥ 0.41 m/s	19	6	25	19/30 (63)	14/20 (70)	19/25 (76)	14/25 (56)	33/50 (66)
	< 0.41 m/s	11	14	25					
6MWT	≥ 126.5 m	18	8	26	18/30 (60)	12/20 (60)	18/26 (69)	12/24 (50)	30/50 (60)
	< 126.5 m	12	12	24					
TUG	≤ 23.11 s	18	5	23	18/30 (60)	15/20 (75)	18/23 (78)	15/27 (56)	33/50 (66)
	> 23.11 s	12	15	27					
F8WT	≤ 23.48 s	17	5	22	17/30 (57)	15/20 (75)	17/22 (77)	15/28 (54)	32/50 (64)
	> 23.48 s	13	15	28					
BBS	≥ 42.5 score	23	6	29	23/30 (77)	14/20 (70)	23/29 (79)	14/21 (67)	37/50 (74)
	< 42.5 score	7	14	21					
mFSST	≤ 32.88 s	21	6	27	21/30 (70)	14/20 (70)	21/27 (78)	14/23 (61)	35/50 (70)
	> 32.88 s	9	14	23					

Se: sensitivity; Sp: specificity; PPV: Positive Predictive Value; NPV: Negative Predictive Value; Ac: accuracy; FAC: Functional Ambulation Category; MBI: Modified Barthel Index; 10mWT: 10-meter Walk Test; 6MWT: 6-minute Walk Test; 5-Times STST: Five-Times Sit-to-Stand Test; TUG: Timed Up and Go test; F8WT: Figure-of-8 Walk Test; BBS: Berg Balance Scale; mFSST: Modified Four Square Step Test.

For classification of independent ambulation on discharge (FAC ≥ 4), BBS and mFSST demonstrated the highest overall classification performance. The BBS cut-off value of ≥ 40.5 points yielded a sensitivity of 92%, specificity of 71%, PPV of 77%, NPV of 89%, and an overall accuracy of 82%. The mFSST cut-off value of ≤ 31.52 s also demonstrated high classification performance, with a sensitivity of 69%, specificity of 92%, PPV of 90%, NPV of 73%, and an accuracy of 80%. In contrast, the classification accuracy of 10mWT, 6MWT, TUG, and F8WT ranged from 62% to 70%.

**Fig. 1:**
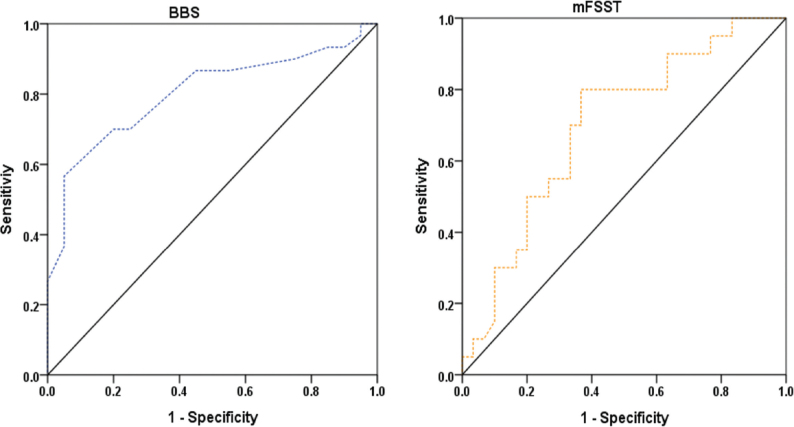
Receiver operating characteristic curves for Berg Balance Scale (BBS) and modified Four Square Step Test (mFSST) predicting independent ambulation (FAC ≥ 4) on discharge.

**Fig. 2:**
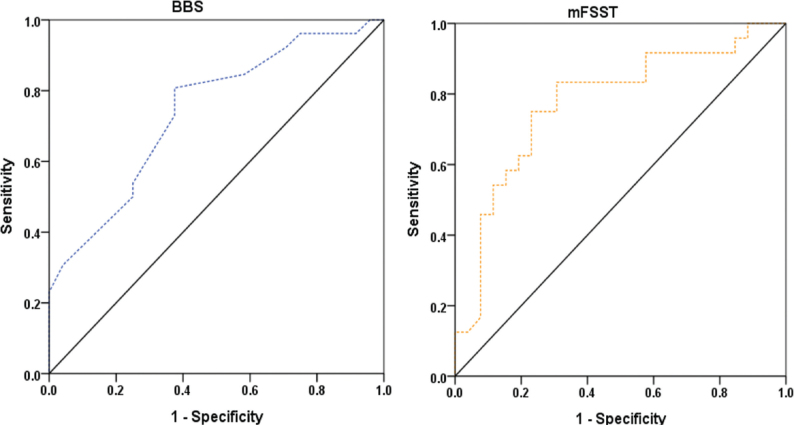
Receiver operating characteristic curves for Berg Balance Scale (BBS) and modified Four Square Step Test (mFSST) predicting functional independence (Modified Barthel Index ≥ 75) on discharge.

For classification of functional independence on discharge (MBI ≥ 75), BBS and mFSST again demonstrated the highest classification performance. The BBS cut-off value of ≥ 42.5 points yielded a sensitivity of 77%, specificity of 70%, PPV of 79%, NPV of 67%, and an accuracy of 74%. The mFSST cut-off value of ≤ 32.88 s yielded a sensitivity of 70%, specificity of 70%, PPV of 78%, NPV of 61%, and accuracy of 70%. In contrast, the classification accuracy of 10mWT, TUG, 6MWT, and F8WT ranged from 60% to 66% (see Table V) (Figs 1 and 2).

## DISCUSSION

This retrospective cohort study compared several admission mobility assessments in subacute stroke inpatients capable of supervised walking. When overall accuracy and the balance between sensitivity and specificity were jointly considered, BBS and mFSST demonstrated the most favourable discriminative performance for both discharge independent ambulation (FAC ≥ 4) and functional independence (MBI ≥ 75). Admission BBS and mFSST scores also demonstrated the strongest associations with discharge FAC and MBI, and their ROC-based performance exceeded that of other commonly used assessments.

Taken together, the findings suggest that early measures capturing dynamic balance and multidirectional stepping are closely aligned with discharge-level independence in patients initially classified as FAC level 3. Relative to gait speed- or time-based single-task measures, BBS and mFSST incorporate postural control, weight shifting, and directional change, which may more closely correspond to the mobility demands underlying FAC and MBI outcomes. From a construct perspective, BBS includes a broad range of postural control tasks and has been described as structurally similar to mobility- and transfer-related subitems of the MBI (15). The mFSST requires rapid directional changes with continuous weight transfer and has been described as capturing reactive balance control and obstacle avoidance ability (30), with reported associations with fall risk and functional mobility. Prior work has also indicated relatively low floor and ceiling effects, supporting its potential utility for discriminating functional levels among individuals with stroke (16, 31, 32). Nevertheless, these explanations are based on existing literature and conceptual considerations rather than mechanisms directly examined in the present study.

The ROC analyses were consistent with this interpretation. For discharge independent ambulation, BBS and mFSST achieved overall accuracies of 82% and 80%, respectively, whereas for discharge functional independence the corresponding accuracies were 74% and 70%. Although several other measures met, or approached, the commonly used threshold for acceptable discrimination (AUC ≥ 0.70) (29), their lower overall accuracy suggests limited usefulness as stand-alone tools for classifying discharge-level independence in this specific cohort.

Importantly, this does not diminish the clinical relevance of gait performance measures. The 6MWT was significantly associated with both discharge outcomes, whereas the 10mWT was associated only with independent ambulation. However, their discriminative performance was modest, with AUC values around the threshold for clinically acceptable discrimination. Because all participants were FAC level 3 on admission, the cohort was relatively homogeneous, which may have attenuated between-group differences in speed and endurance. Accordingly, gait speed and endurance based assessments may remain useful as supplementary indicators of specific mobility components but may be insufficient to discriminate independent ambulation or functional independence on discharge when used in isolation.

Similar considerations apply to time-based mobility tests. The 10mWT, TUG, and F8WT primarily quantify speed or task completion time and may have limited capacity to represent complex balance strategies or multidirectional mobility demands. In the present study, their correlations with discharge outcomes were smaller than those observed for BBS and mFSST. For the 6MWT, feasibility constraints in subacute inpatients – such as reduced physical capacity, fatigue, and environmental requirements – may limit routine implementation (33, 34). Consistent with this, a clinical survey reported a relatively low utilization rate of the 6MWT (approximately 11%) in subacute stroke inpatients (10, 22). The 5-Times STST, which largely reflects lower-extremity strength during a transitional task, also demonstrated limited discriminative performance in the present study (24) and has been reported to correlate weakly with composite balance indices such as BBS and limits of stability measures (24). Overall, the present findings are broadly consistent with previous studies. BBS has been reported to correlate strongly with FAC (*r* = 0.865), the Functional Independence Measure (*r* = 0.72 to 0.81) (35, 36), and the Barthel Index (*r* = 0.76 to 0.91) (37, 38), supporting its role as a core assessment reflecting functional status in subacute stroke. For mFSST, direct evidence regarding relationships with FAC and MBI remains limited; however, strong correlations with BBS (*r* = −0.62 to −0.87) have been reported (16, 39, 40). The comparable association observed in the present study (*r* = −0.61 to −0.63) further supports the interrelatedness of these measures and is consistent with the view that mFSST may complement BBS as a clinically useful screening tool for discharge-level independence.

Compared with earlier work, this study aimed to provide more practice-oriented evidence by reporting a broader set of performance indices. Shim (17) used admission BBS (38.5 points) and lower-extremity Fugl-Meyer Assessment scores (29.5 points) to discriminate discharge independent ambulation (FAC ≥ 4) in subacute stroke inpatients, but reported only sensitivity and specificity, limiting comprehensive appraisal of clinical utility. In contrast, the present study compared multiple assessments spanning gait speed, endurance, balance, strength, and multidirectional mobility, and reported sensitivity, specificity, PPV, NPV, accuracy, and ROC-derived cut-off values intended for clinical interpretation. In line with the exploratory design, these cut-offs should be interpreted as screening thresholds rather than definitive criteria.

Several limitations should be acknowledged. First, the retrospective single-centre EMR-based design limits representativeness and warrants caution in generalization. Second, although assessments were standardized within 3 days of admission and approximately 4 weeks later, time since stroke onset ranged from 2 to 5 months and may not fully capture recovery-stage heterogeneity. Third, multicollinearity among variables and the number of candidate predictors relative to the sample size precluded stable multivariable modelling; therefore, analyses focused on exploratory univariable comparisons and ROC-based indices.

Fourth, restricting the cohort to patients with admission FAC level 3 improves clinical relevance by targeting a transitional subgroup in whom discharge-level classification is particularly challenging, but it limits extrapolation to the broader subacute stroke population. Although this criterion may appear to select patients primarily limited by balance and coordination, FAC level 3 represents a heterogeneous transitional stage of ambulation recovery in which motor function, balance, and gait capacity interact. In our cohort, functional assessments indicated limitations not only in balance but also in lower-limb motor function, walking performance, and multidirectional mobility. Patients with FAC ≤ 2 were not included because gait-related assessments cannot be performed consistently at this level of required assistance, which could increase measurement error and complicate interpretation of gait performance outcomes. In addition, cut-off values were derived and evaluated in the same dataset, introducing potential optimism bias. Although internal validation methods such as cross-validation or bootstrap testing could improve stability and generalizability, they were not performed due to the relatively small sample size. Future studies with larger cohorts should include internal validation procedures.

These findings support the clinical importance of multidimensional mobility assessment incorporating balance and multidirectional stepping ability to inform early goal setting and discharge planning. External validation in multicentre prospective studies is warranted to confirm the generalizability and clinical applicability of the proposed cut-off values and performance indices.
